# The prevalence of stress, anxiety and depression within front-line healthcare workers caring for COVID-19 patients: a systematic review and meta-regression

**DOI:** 10.1186/s12960-020-00544-1

**Published:** 2020-12-17

**Authors:** Nader Salari, Habibolah Khazaie, Amin Hosseinian-Far, Behnam Khaledi-Paveh, Mohsen Kazeminia, Masoud Mohammadi, Shamarina Shohaimi, Alireza Daneshkhah, Soudabeh Eskandari

**Affiliations:** 1 Department of Biostatistics, School of Health, Kermanshah University of Medical Sciences, Kermanshah, Iran; 2 Sleep Disorders Research Center, Kermanshah University of Medical Sciences, Kermanshah, Iran; 3 Department of Business Systems & Operations, University of Northampton, Northampton, UK; 4 Department of Nursing, School of Nursing and Midwifery, Kermanshah University of Medical Sciences, Kermanshah, Iran; 5 Department of Biology, Faculty of Science, University Putra Malaysia, Serdang, Selangor Malaysia; 6 School of Computing, Electronics and Maths, Coventry University, London, UK

**Keywords:** Anxiety, Stress, Depression, COVID-19, Healthcare workers

## Abstract

**Background:**

Stress, anxiety, and depression are some of the most important research and practice challenges for psychologists, psychiatrists, and behavioral scientists. Due to the importance of issue and the lack of general statistics on these disorders among the Hospital staff treating the COVID-19 patients, this study aims to systematically review and determine the prevalence of stress, anxiety and depression within front-line healthcare workers caring for COVID-19 patients.

**Methods:**

In this research work, the systematic review, meta-analysis and meta-regression approaches are used to approximate the prevalence of stress, anxiety and depression within front-line healthcare workers caring for COVID-19 patients. The keywords of prevalence, anxiety, stress, depression, psychopathy, mental illness, mental disorder, doctor, physician, nurse, hospital staff, 2019-nCoV, COVID-19, SARS-CoV-2 and Coronaviruses were used for searching the SID, MagIran, IranMedex, IranDoc, ScienceDirect, Embase, Scopus, PubMed, Web of Science (ISI) and Google Scholar databases. The search process was conducted in December 2019 to June 2020. In order to amalgamate and analyze the reported results within the collected studies, the random effects model is used. The heterogeneity of the studies is assessed using the *I*^2^ index. Lastly, the data analysis is performed within the Comprehensive Meta-Analysis software.

**Results:**

Of the 29 studies with a total sample size of 22,380, 21 papers have reported the prevalence of depression, 23 have reported the prevalence of anxiety, and 9 studies have reported the prevalence of stress. The prevalence of depression is 24.3% (18% CI 18.2–31.6%), the prevalence of anxiety is 25.8% (95% CI 20.5–31.9%), and the prevalence of stress is 45% (95% CI 24.3–67.5%) among the hospitals’ Hospital staff caring for the COVID-19 patients. According to the results of meta-regression analysis, with increasing the sample size, the prevalence of depression and anxiety decreased, and this was statistically significant (*P* < 0.05), however, the prevalence of stress increased with increasing the sample size, yet this was not statistically significant (*P* = 0.829).

**Conclusion:**

The results of this study clearly demonstrate that the prevalence of stress, anxiety and depression within front-line healthcare workers caring for COVID-19 patients is high. Therefore, the health policy-makers should take measures to control and prevent mental disorders in the Hospital staff.

## Background

On 31st December 2019, China reported an acute pneumonia outbreak that had emerged from Wuhan. In a short span of time, the disease caused by the new coronavirus (COVID-19) spread from China to other countries, and caused several health, socio-economic and political challenges globally [1, 2]. On 30th January 2020, the World Health Organization (WHO) declared the 2019 New Coronavirus as a Public Health Emergency of International Concern (PHEIC). On February 11, 2020, WHO declared a global pandemic, and officially named the new coronavirus as COVID-19 [2–4]. On the same day, the International Committee on Virus Classification (ICTV) modified the 2019-nCoV name to SARS-CoV-2 [4]. As of June 25, 2020, the WHO reported more than 8.5 million infections worldwide. Nevertheless, the number of the infected people is still increasing. Moreover, the lack of a definitive treatment has led to more than 457,000 fatalities during this period [4, 5]. The outbreak of the disease has put a lot of psychological pressure on different communities and keyworkers, especially Hospital staff who are in a direct contact with the patients [5].

Stress, anxiety, and depression are some of the key challenges for psychologists, psychiatrists, and behavioral scientists globally. Among physical and mental illnesses, depression is common mental disorder in the world depression [6], according to the World Health Organization, is one of the most common behavioral disorders associated with low mood, loss of interest, guilt and worthlessness, sleep and appetite disorders, decreased energy and decreased concentration. Depression and anxiety are the most common psychiatric disorders with a prevalence of 10 to 20% in the general population [6–9]. Stress is in fact an integral part of human life and is perhaps one of the most common issues in modern societies [6, 11]. Anxiety is a disorder often associated with fear and unease and is accompanied by symptoms such as fatigue, restlessness and palpitations. In the etiology of anxiety, genetic, hereditary, environmental, psychological, social and biological factors are considered [6, 12, 13]. A person who is exposed to constant anxiety and worry loses self-confidence and becomes depressed while feeling humiliated, and these in turn increase workplace stress and performance reduction. The latter itself intensifies anxiety, and the continuation of this cycle can eventually erode people's mental and physical abilities and, after a while, lead to unstable neuropsychiatric disorders [6, 14].

Nurses and physicians are affected by a variety of stressors in their workplaces because of their responsibility to provide health and treatment to patients, the National Institutes of Health (NIH) said after studying the relative prevalence of health disorders in high-stress occupations. Out of 130 jobs surveyed, nursing is ranked 27th due to mental health problems [15]. Other studies report that 7.4% of nurses are absent from work each week due to burnout or disability due to stress, which is 80% more than other occupational groups [15].

Hospital Hospital staff in charge of admitting and caring for patients with COVID-19 have been subjected to a variety of individual, and organizational stresses that have adversely affected their health and job satisfaction. Therefore, recognizing stressors, and periodic training will be an effective step towards prevention, treatment and stress reduction [10–14]. Stress can increase depression and anxiety, reduce job satisfaction, impair individual relationships, and even lead to suicidal thoughts. It can also reduce the effects of psychological interventions due to the reduction in concentration and decision-making skills, and by influencing the mental health professional's ability to communicate strongly with clients [15].

Due to the impact of various factors on the prevalence of stress, anxiety and depression in hospitals’ Hospital staff directly faced with the COVID-19 patients, and the lack of general statistics in this regard, we attempted to systematically review the literature. We statistically analyzed the reported results of the collected studies to provide a set of general statistics on the prevalence of stress, anxiety and depression within front-line healthcare workers caring for COVID-19 patients, with a view to inform other related programs for reducing the complications of these disorders.

## Methods

This work has followed the systematic review, meta-analysis, and meta-regression methods. In order to identify relevant studies from literature the SID, MagIran, IranMedex, IranDoc, ScienceDirect, Embase, Scopus, PubMed, Web of Science (ISI) and Google Scholar databases were searched. The keywords of prevalence, anxiety, stress, depression, psychopathy, mental illness, mental disorder, doctor, physician, nurse, Hospital staff, 2019-nCoV, COVID-19, SARS-CoV-2 and Coronaviruses and all possible combinations of these words were used in the search strategy and for each of the above-mentioned databases. No lower time limit was considered in the search process, and articles published in December 2019 to June 2020 were among the search pool. Once all related studies were identified, the identifying information about the selected sources was transferred into the EndNote bibliography management software. In order to maximize the comprehensiveness of the search, the reference lists within all selected articles were manually reviewed.

### Inclusion criteria

Criteria for entering studies included: studies examining the prevalence of stress, anxiety, and depression in the hospital Hospital staff caring for COVID-19 patients based on the diagnostic criteria in each study (SDS, SAS, SASR, DASS-21, BDI-II, BAI, PSS, HAD, GAD-7) (Table 1).

**Table 1:** Characteristic of the collected studies related to the prevalence of depression, anxiety and stress

Author, year, references	Age (years)	Country	Sample size			Prevalence%			Mean ± standard deviation			Diagnostic criteria	Population
			Total	Female	Male	Depression	Anxiety	Stress	Depression	Anxiety	Stress		
Zhu-1, 2020 [7]	34.16 ± 8.06	China	79	–	–	45.6	11.4	–	–	–	–	SDS^a^ SAS^b^	Doctor
Zhu-2, 2020 [7]	–	China	86	–	–	43	27.9	–	–	–	–	SDS SAS	Nurse
Xiao-1, 2020 [8]	–	China	180	129	51	–	29.4	29.4	–	55.25 ± 14.18	77.58 ± 29.52	SAS SASR^c^	Hospital staff
Chew, 2020 [9]	29 (25–35)	Singapore	906	583	323	10.6	15.7	5.2	–	–	–	DASS-21^d^	Hospital staff
Zhang-1, 2020 [10]	–	Hong Kong	564	478	77	39.5	30.0	–	–	–	–	DASS-21	Doctor
Zhang-2, 2020 [10]	–	Hong Kong	999	804	195	31.0	25.4	–	–	–	–	DASS-21	Nurse
Wang-1, 2020 [11]	–	China	194	–	–	–	–	–	6.25 ± 1.93	6.16 ± 2.22	7.76 ± 15.2	BDI-II^e^ BAI^f^ PSS^g^	Doctor
Wang-2, 2020 [11]	–	China	1304	–	–	–	–	–	6.38 ± 1.66	6.15 ± 2.36	7.86 ± 17.5	BDI-II BAI PSS	Nurse
Liu-1, 2020 [13]	–	China	512	433	79	–	12.5	–	–	39.56 ± 8.91	–	SAS	Hospital staff
Du, 2020 [17]	36.00 ± 8.05	China	134	81	53	12.7	20.1	–	5.76 ± 7.04	4.96 ± 8.13	–	BDI-II BAI	Hospital staff
Kazmi, 2020 [18]	–	Iran	1000	620	380	61.1	57.0	64.3	–	–	–	DASS-21	Hospital staff
Gautam, 2020 [19]	–	China	187	–	–	–	23.0	–	–	–	–	SAS	Hospital staff
Ong, 2020 [20]	–	China, Indian, Others	158	111	47	0.6	0.6	–	–	–	–	DASS-21	Hospital staff
Xiao-2, 2020 [21]	–	China	958	644	314	42.7	39.9	–	–	–	–	HAD^h^	Hospital staff
Guixia, 2020 [22]	–	China	86	–	–	54.7	44.2	45.3	5.78 ± 4.36	46.89 ± 10.47	50.7 ± 12.11	BDI-II SAS SASR	Hospital staff
Aghili, 2020 [23]	–	Iran	289	–	–	44.6	33.9	71.3	–	–	–	SDS SAS SASR	Hospital staff
Dimitriu, 2020 [24]	27.92 ± 2.66	Romania	100	50	50	9.0	–	–	–	–	–	SDS SAS	Hospital staff
Sharif, 2020 [25]	–	India	375	–	–	13.9	–	–	–	–	–	SDS	Hospital staff
Geoffroy, 2020 [26]	32.7 ± 11.00	France	149	–	–	4.0	49.0	70.5	–	–	–	DASS-21	Hospital staff
Abdulah, 2020 [27]	35.06 (33–70)	Australia	268	80	188	–	–	93.7	–	–	4.20 ± 2.46	PSS	Doctor
Yin, 2020 [28]	35.30 ± 9.48	China	371	228	143	–	–	44.5	–	–	–	SASR	Hospital staff
Gao, 2020 [29]	–	China	118	88	30	44.9	44.9	–	–	–	–	SDS SAS	Hospital staff
Huang-1, 2020 [30]	–	China	230	187	43	–	23.0	–	–	–	–	SAS	Hospital staff
Huang-2, 2020 [31]	49(41–58)	China	2250	1642	608	19.8	35.6	–	–	–	–	GAD-7^i^	Hospital staff
Lai, 2020 [32]	26–40	China	1257	964	293	50.4	44.6	–	–	–	–	GAD-7	Hospital staff
Liu-2, 2020 [33]	–	China	4679	3851	828	34.6	16.0	–	–	–	–	SDS SAS	Nurse
Lu, 2020 [34]	–	China	2299	1779	512	11.7	24.7	–	–	–	–	SDS SAS	Nurse
Tan, 2020 [35]	–	China	470	321	149	8.9	14.5	6.6	–	–	–	DASS-21	Hospital staff
Zhang-3, 2020 [36]	–	China	2178	1401	781	10.7	10.5	–	–	–	–	SDS SAS	Hospital staff

^a^Self-rating Depression Scale (SDS)

^b^Self-rating Anxiety Scale (SAS)

^c^Stanford Acute Stress Reaction (SASR)

^d^Depression Anxiety Stress Scales (DASS-21)

^e^Beck Depression Inventory-II (BDI-II)

^f^Beck Anxiety Inventory (BAI)

^g^Perceived Stress Scale (PSS)

^h^Hospital Anxiety/Depression Scale (HAD)

^i^The 7-item Generalized Anxiety Disorder (GAD-7) Scale (range, 0–21)

### Exclusion criteria

Criteria for excluding a study were: research works without sufficient data, duplicate papers, and studies with unclear methods (diagnostic methods other than those listed in the inclusion criteria).

### Study selection

Initially, studies that were repeated in various databases were removed from the list. Subsequently, a list of the titles of all the remaining articles was prepared, so that the quality of articles could be evaluated. For the systematic review, the PRISMA guidelines were followed; in the first stage, screening, the title and abstract of the remaining articles were carefully examined and a number of irrelevant articles were excluded, considering the inclusion and exclusion criteria. In the second stage, i.e., eligibility evaluation, the full text of the possible related articles remaining from the screening stage were examined, and similarly, at this stage, several other irrelevant studies were removed. To prevent bias, all stages of resource review and data extraction were performed by two reviewers independently. If an article was not included, the reason for the exclusion was mentioned. In cases where there was a disagreement between the two reviewers, the third person reviewed the article.

### Quality evaluation of articles

In order to evaluate the quality of articles (i.e., with respect to the methodological validity and results), a checklist appropriate to the type of study was used. The STROBE checklists are commonly used to critique and evaluate the quality of observational studies. The Strengthening the Reporting of Observational studies in Epidemiology (STROBE checklist) consists of six scales/general sections that include: title, abstract, introduction, methods, results, and discussion. Some of these scales have subscales, resulting in a total of 32 subscales (items). Some of these 32 items represent different methodological aspects of the study, and include title, problem statement, study objectives, study type, study statistical community, sampling strategy, sample size, definition of variables and procedures, data collection tools, statistical analysis methods, and findings. Accordingly, the maximum score that can be obtained from the evaluation using the checklist is 32. Considering the score of 16 as the cut-off point [16], all articles with scores of 16 and above were considered as medium or high-quality articles. Sixteen articles were considered as low quality, and were therefore excluded from the study.

### Data extraction

Information on all final papers entered into the systematic review, meta-analysis, and meta-regression process were extracted using another pre-prepared checklist. The checklist included the title of the article, the name of the first author, the year of publication, the place of study, the study population, the research instrument, the sample size, the prevalence of stress, anxiety and depression.

### Statistical analysis

To assess the heterogeneity of the selected articles, the *I*^2^ index was used [heterogeneity was considered in three categories: less than 25% (low heterogeneity), 25–75% (medium heterogeneity), and more than 75% (high heterogeneity)]. In order to investigate the publication bias and also due to the high volume of samples entered in the study, Begg's test (Begg and Mazumdar) was performed at the significance level of 0.1, and the corresponding Funnel plots were included. In this study, in order to investigate the factors affecting the heterogeneity of studies, meta-regression analysis was used to investigate the effect of the sample size on meta-analysis. Data analysis was performed using the Comprehensive Meta-Analysis (version 2) software.

## Results

**Fig. 1:**
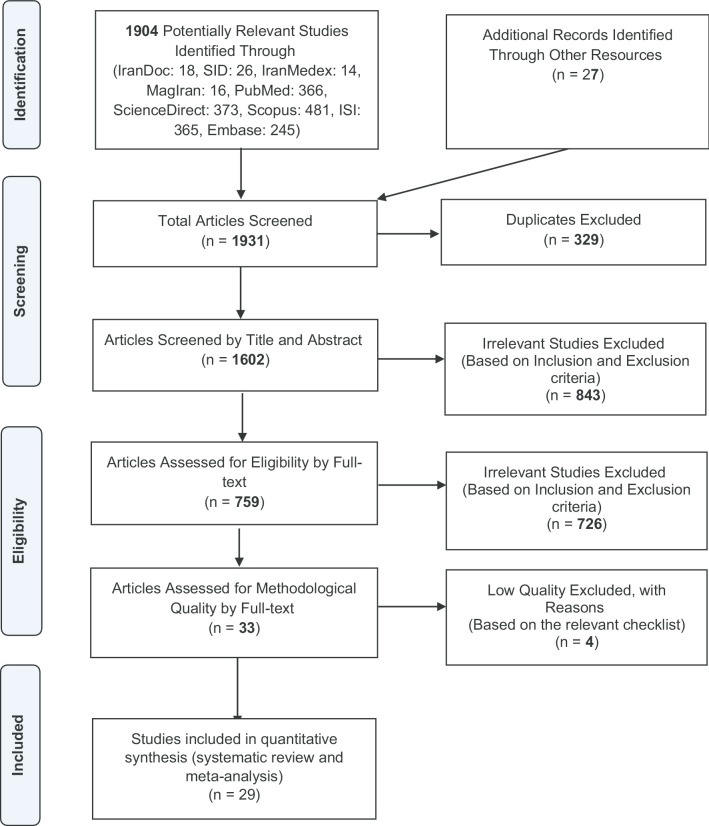
The flowchart on the stages of including studies in the systematic review and meta-analysis (PRISMA 2009)

As mentioned earlier, the Preferred Reporting Items for Systematic Reviews and Meta-Analysis (PRISMA guidelines) were used to conduct the systematic review, meta-analysis, and the meta-regression. At the identification stage, 1904 possible related articles were identified and transferred into the EndNote bibliography management software. Another 27 studies were included following the examination of list of sources and gray literature. Of the total 1931 studies identified, 329 were duplicate and were therefore excluded. In the screening stage, of the 1602 remaining studies, 843 articles were omitted by studying their title and abstract and based on the inclusion and exclusion criteria. In the evaluation eligibility stage, out of 759 remaining articles, 726 ineligible articles were removed following the examination of their full text, and similarly according to the inclusion and exclusion criteria. In the quality evaluation stage, by reading the full text of the article and based on the score obtained from STROBE checklist, out of 33 remaining studies, four studies were assessed as low quality and excluded (i.e., STROBE checklist score below 16). Finally, 29 articles that are published until June 2020 were entered into the final analysis (Fig. 1).

The *I*^2^ test results for depression, anxiety, and stress were 98.9, 98.5, and 99.1, respectively. Due to the heterogeneity of the selected studies, the random effects model was used to amalgamate the reported results and to estimate the overall prevalence of each disorder. The reason for the heterogeneity between studies can be due to different sample size, sampling error, study time, or study location. Of the 29 studies with the total sample size of 22,380, 21 studies had a focus on depression, 23 studies reported anxiety, and 9 articles studied stress in Hospital staff caring for the COVID-19 patients. The lowest and highest sample sizes were related to the studies of Zhu et al. [7] (79 participants), and Liu et al. [33] (4679 participants), respectively. The specifications of the meta-analysis studies are provided in Table 1.

The publication bias in reporting the results of the prevalence of depression, anxiety, and stress using funnel diagrams and Begg’s tests at the significance level of 0.1 indicates no bias in the present study (*P* = 0.349, *P* = 0.711, and *P* = 0.916, respectively).

**Fig. 2:**
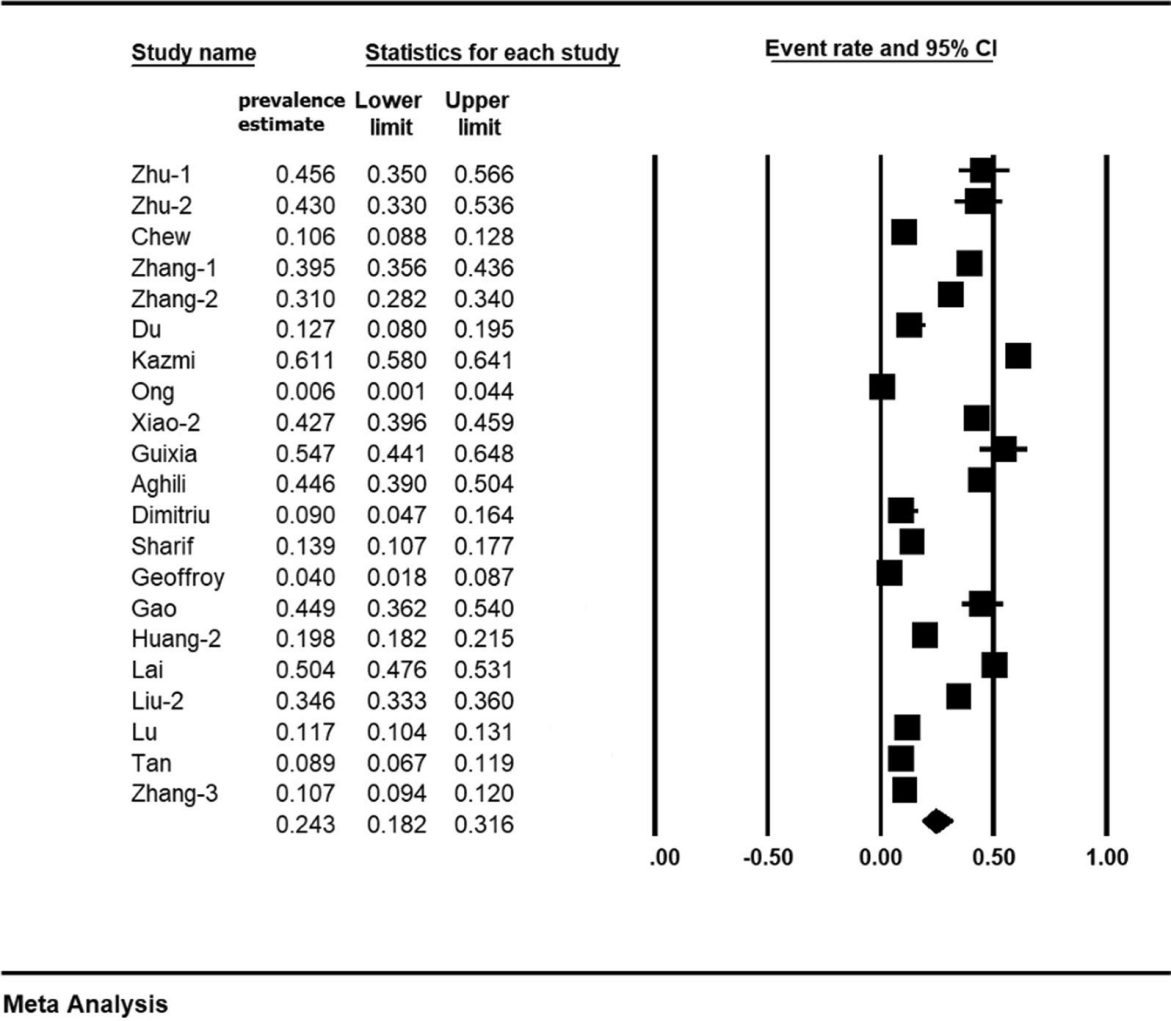
Forest plot demonstrating the prevalence of depression within front-line healthcare workers caring for COVID-19 patients; 95% CI

**Fig. 3:**
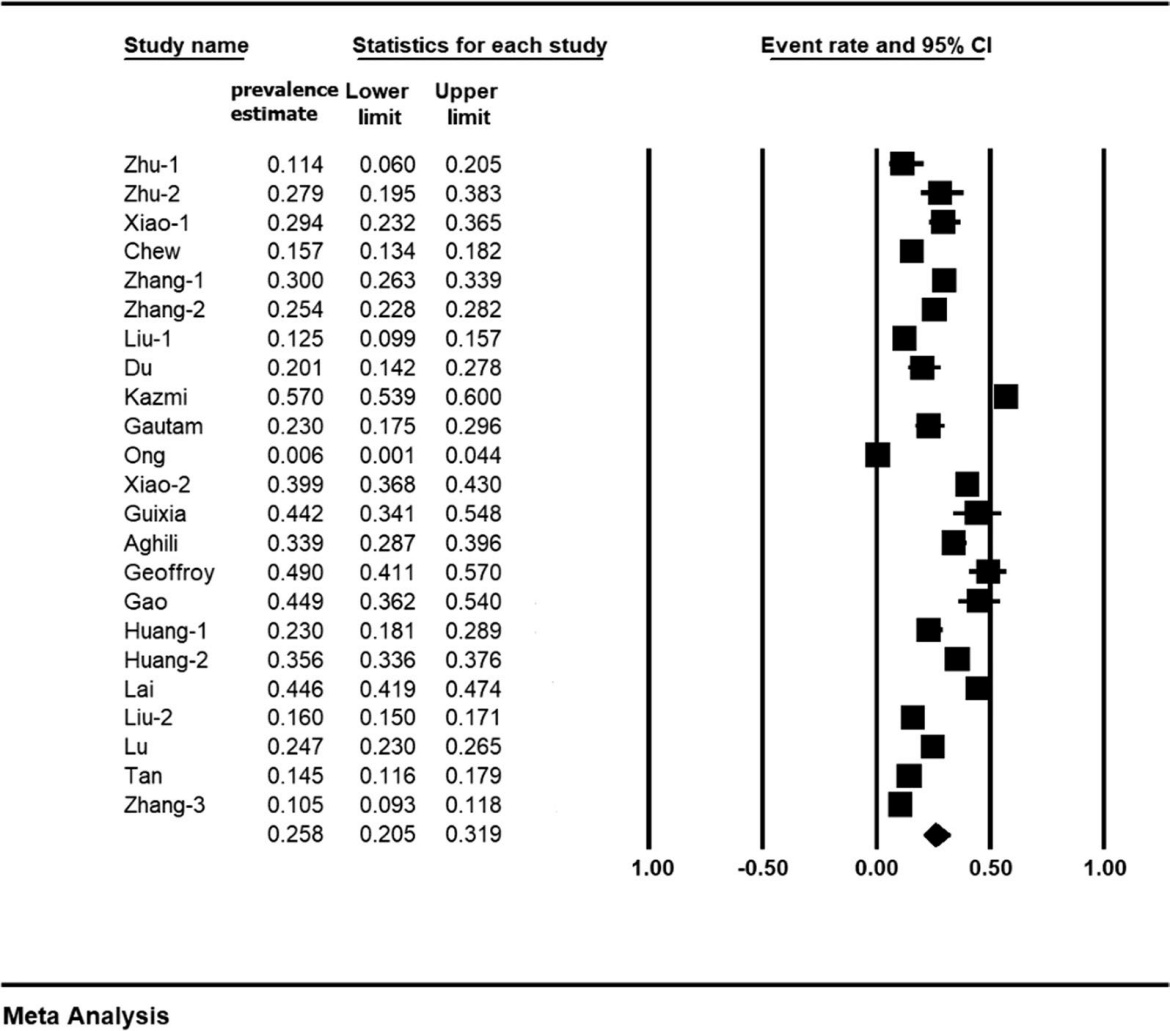
Forest plot demonstrating the prevalence of anxiety within front-line healthcare workers caring for COVID-19 patients; 95% CI

**Fig. 4:**
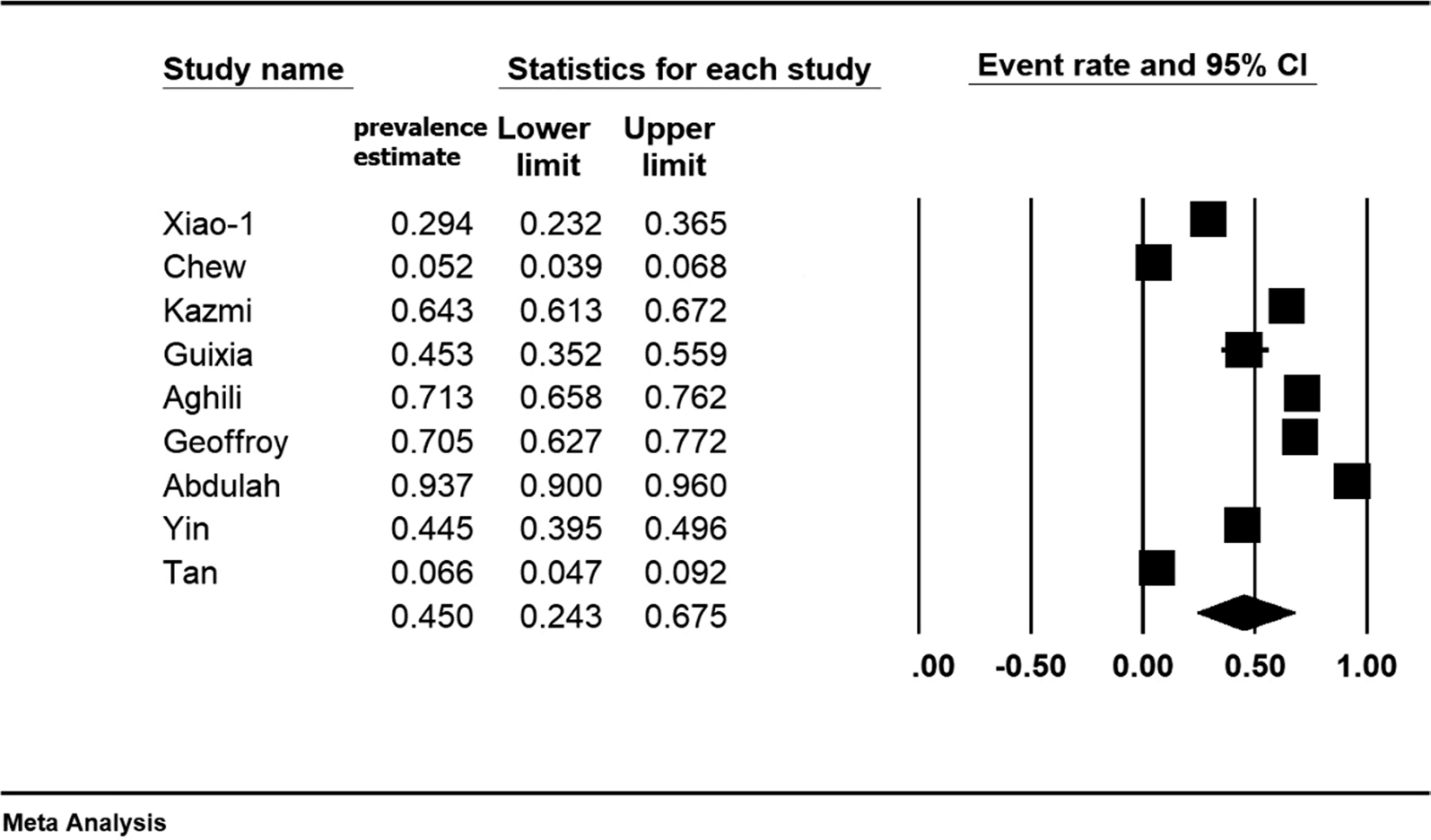
Forest plot demonstrating the prevalence of stress within front-line healthcare workers caring for COVID-19 patients; 95% CI

Our findings show that the prevalence of depression is 24.3% (95% CI 18.2–31.6%), the prevalence of anxiety is 25.8% (95% CI 20.5–31.9%), and the prevalence of stress is 45% (95% CI 24.3–67.5%) in the hospital Hospital staff caring for the COVID-19 patients. The midpoint of each square in the following forest plots indicates ‘prevalence’ in each study, and the diamond shape denotes the overall prevalence in the total population and for all studies combined (Figs. 2, 3 and 4).

### Meta-regression test

**Fig. 5:**
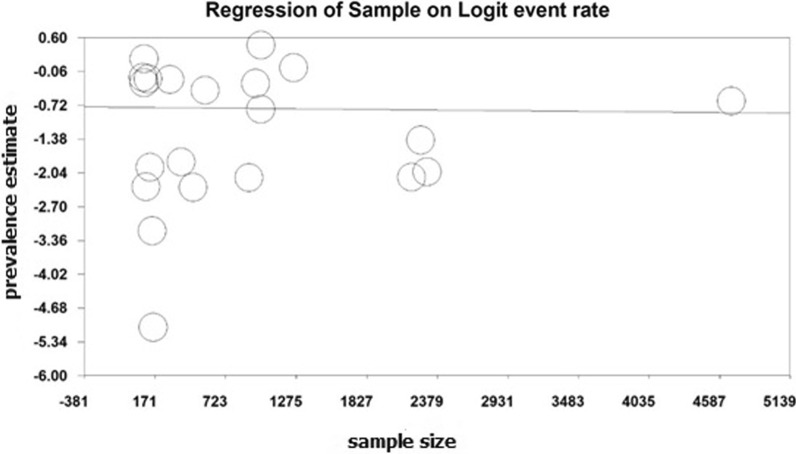
Meta-regression chart of the prevalence of depression by sample size

**Fig. 6:**
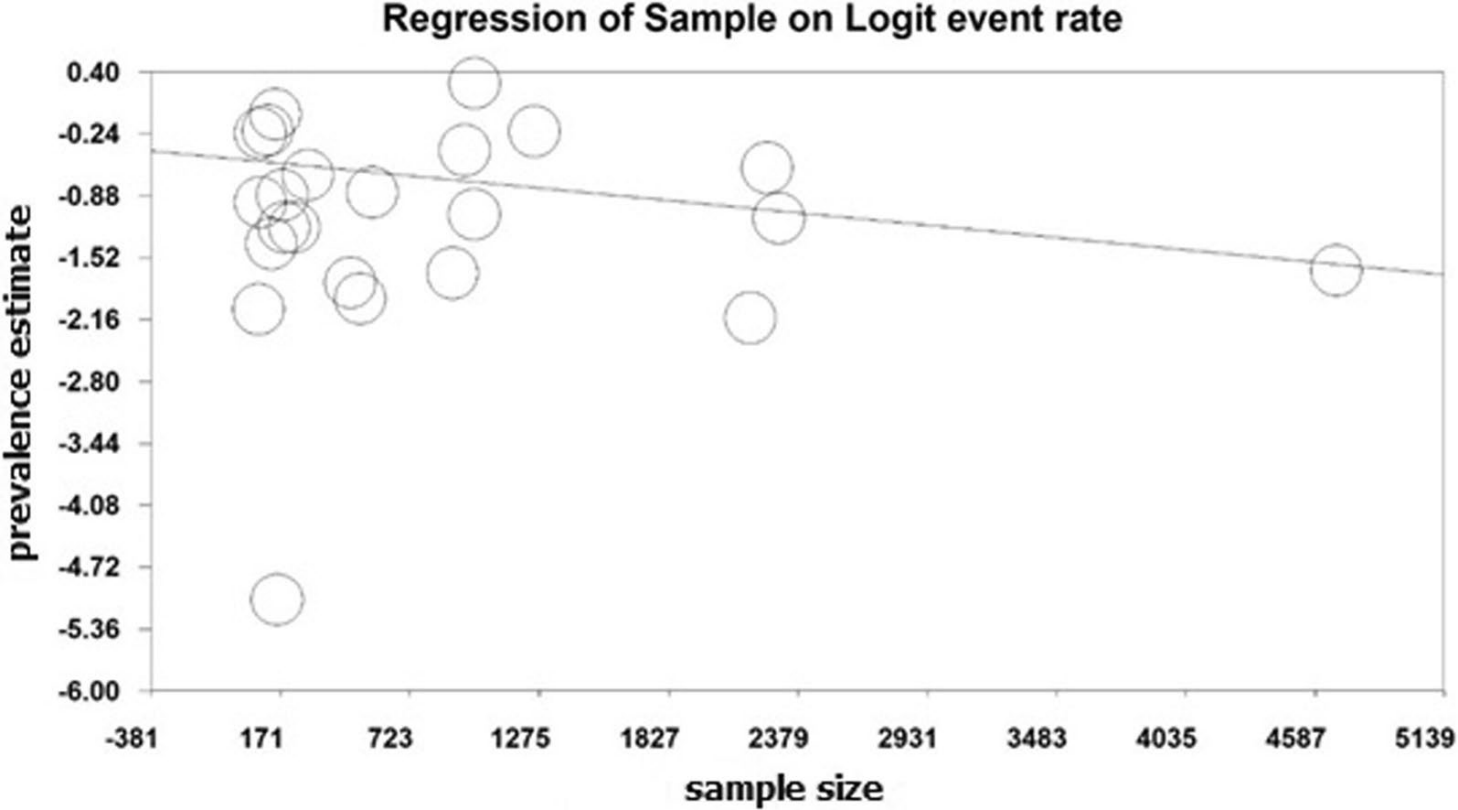
Meta-regression chart of the prevalence of anxiety by sample size

**Fig. 7:**
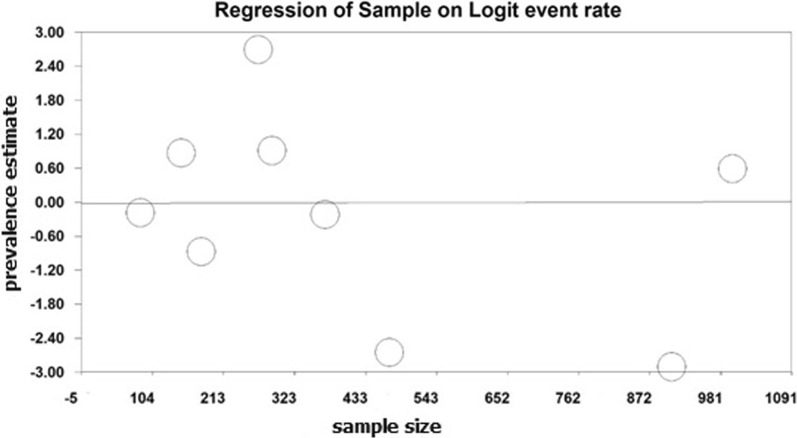
Meta-regression chart of the prevalence of stress by sample size

In order to investigate potential factors affecting the heterogeneity of depression, anxiety and stress prevalence, and to assess study effect size, meta-regression technique was used (Figs. 5, 6 and 7). According to Fig. 5, the prevalence of depression decreases with increasing sample size, and this is statistically significant (*P* < 0.05). Considering Fig. 6, increasing the sample size, decreases the prevalence of anxiety, which is statistically significant (*P* < 0.05). Moreover, according to Fig. 7, there was no significant relationship between sample size and the prevalence of stress (*P* = 0.829).

### Subgroup analysis based on the type of job of the hospital’s Hospital staff

Considering the results presented in Table 2, in Hospital staff other than physicians and nurses, the prevalence of depression is 20.6% (95% CI 13.1–30.9%), the prevalence of anxiety is 27% (95% CI 20.1–35.3%), and the prevalence of stress is 36.4% (95% CI 18.3–59.5%). Moreover, in physicians, the prevalence of depression is 40.4% (95% CI 36.4–44.5%), the prevalence of anxiety 19.8% (95% CI 7.1–44.3%), and the prevalence of stress is 93.7% (95% CI 90–96%). Furthermore, the prevalence of depression, and anxiety in nurses is 28% (95% CI 16–44.2%). and 22.8% (95% CI 17–29.8%), respectively (Table 2).

**Table 2:** Subgroup analysis

Hospital medical staff	Type of disorder	Number of articles	Sample size	*I*^2^	Begg and Mazumdar	Prevalence (95% CI)
Hospital staff (non-physicians and nurses)	Depression	15	10,658	99.1	0.317	20.6 (95% CI 13.1–30.9)
	Anxiety	17	11,062	95.5	0.258	27 (95% CI 20.1–35.3)
	Stress	8	3551	99	0.180	36.4 (95% CI 18.3–59.5)
Physicians	Depression	2	643	4.2	–	40.4 (95% CI 36.4–44.5)
	Anxiety	2	643	90.7	–	19.8 (95% CI 7.1–44.3)
	Stress	1	268	0	–	93.7 (95% CI 90–96)
Nurses	Depression	4	8063	99.2	0.667	28 (95% CI 16–44.2)
	Anxiety	4	8063	96.5	0.514	22.8 (95% CI 17–29.8)

Accordingly, it is reported that the prevalence of depression in physicians is much higher than nurses and Hospital staff, and the prevalence of anxiety in Hospital staff is much higher than other groups studied. Also, only one study reviewed by Australian physicians shows a much higher prevalence of stress than the results of other studies in nurses and Hospital staff.

## Discussion

The aim of the present study was to conduct a systematic review, meta-analysis and meta-regression, to determine the prevalence of stress, anxiety and depression within front-line healthcare workers caring for COVID-19 patients. According to our findings, the overall prevalence of stress is 45%, and also according to the analysis of subgroups, the prevalence of stress in physicians is higher than other groups of Hospital staff. The highest prevalence of stress was reported in the study of Abdulah et al. [27] with 93.7%, and the lowest prevalence was related to the study of Chew et al. [9] with 5.2%. The most comprehensive study in terms of sample size was related to a research conducted by Kazmi et al. [18] in Iran, who reported the prevalence of stress as 64.3%, among Hospital staff dealing with the COVID-19 patients. Anxiety, depression and stress have been studied in Hospital staff treating other groups of patients. For instance, in the meta-analysis performed by Costello et al. [37], the prevalence of stress in staff caring for patients with dementia was 18.34%, and in the study of Cheung et al. [38], the prevalence of stress in Hong Kong nurses was reported to be 8.73%. A different piece of research conducted by Kulsoom et al. [39] stated that the prevalence of stress in medical students in Saudi Arabia was 30–41%%. The findings of our work demonstrate a higher prevalence of behavioral disorders in Hospital staff caring for the COVID-19 patients. This indicates urgent attention and possible interventions are required by related policy-makers and authorities.

In modern societies, stress at work is an important factor to consider in the healthcare sector [40]. Stress at workplaces raises concerns about people’s mental health [41]. Workplace stress is defined as an emotional, perceptual, behavioral, and physiological response pattern to adverse aspects of work, organization, and the workplace environment [42]. The effect of job stress on physical and mental illness is significant [43]. Job or job-related stresses are undoubtedly one of the leading causes of mental health concerns globally [44]. High levels of stress can impair employees’ performance as well as negatively affect their attitudes and behaviors [45, 46]. Additionally, occupational stress has been shown to impose a cost of 300 to 400 million dollars on healthcare systems [47]. For this reason, identifying the causes and the prevalence of workplace stress among Hospital staff caring for the COVID-19 patients is important, and can help to protect and safeguard the workforce as well as to improve the quality of service provided to patients.

According to our systemic review and meta-analysis, the overall prevalence of anxiety is 25.8%. Considering the subgroups analysis, the prevalence of anxiety in physicians is lower than other Hospital staff, although the CI is wide and the difference is not significant. The highest prevalence of anxiety was related to the study of Kazmi et al. [18] with 57%, and the lowest prevalence was related to the work of Ong et al. [20] with 0.6%. The most comprehensive study in terms of the sample size was conducted by Liu-2 et al. in China [33], which reported a 16% prevalence of anxiety among Hospital staff caring for the COVID-19 patients. The prevalence of anxiety as a disorder has also been assessed in other contexts. For instance, in a meta-analysis conducted by Fawzy et al. [48], the prevalence of anxiety in Egyptian medical students was 73%, and in the study of Cheung et al. [38], prevalence of anxiety in Hong Kong nurses was reported to be 50.1%, which is higher than the current study reporting the prevalence of anxiety. Nevertheless, Kisely et al. [49] reported that the prevalence of anxiety in the general American population was 10.5%, indicating that the prevalence of anxiety in the Hospital staff caring for the COVID-19 patients is higher than in the general population. Such differences may be due to the selection of more specialized keywords in search, review of study quality and evaluation of articles by two researchers in order to prevent the entry of irrelevant articles.

Anxiety is caused by the inability to resolve mental conflicts, and largely parts of a person's mental strength are spent on resolving psychological conflicts. For this reason, people with psychological illnesses are unable to properly use their abilities and talents to the optimum levels. Such psychological contradictions and conflicts, deteriorate their strength and mental energy, and cause inconsistencies in mental investments in all psychological needs and dimensions [50]. The key negative consequences of anxiety include reduced quality of life, inability and greater need for health services and increased mortality. Therefore, early detection and appropriate treatment prevent such consequences [51–53]. Hitherto, various methods have been identified that reduce anxiety, such as support from family and friends, socialization, proper nutrition [54], mental preparation, light exercise, music, psychotherapy [55], adequate rest, use of sleep medicine, prescription of anti-anxiety drugs [54], relaxation training [56], and aerobic exercise [57].

Similarly, according to our findings, the prevalence of depression is 24.3% and also according to the subgroup's analysis, the prevalence of depression in physicians is higher than in other Hospital staff. The highest prevalence of depression was related to a study conducted by Kazmi et al. [18] with 61.1%, and the lowest prevalence was reported in work of Ong et al. [20] with 0.6%. The most comprehensive study in terms of sample size was performed in the research of Liu-2 et al. [33] that reported the prevalence of anxiety among Hospital staff caring for the COVID_19 patients as 34.6%. Research works on the depression prevalence have been done in other contexts as well. For instance, in a meta-analysis conducted by Costello et al. [37], the prevalence of depression in patients with dementia was reported as 6.29%, and in the piece of research by Lei et al. [58], the prevalence of depression in Chinese physicians was reported as 23.8%. Sarokhani et al. [59] demonstrated that the prevalence of depression in Iranian medical students was 23%. Moreover, in a meta-analysis study by Mata et al. [60], the prevalence of depression in physicians was reported as 20.9%. Our study demonstrates that the prevalence of depression in the hospitals’ Hospital staff caring for the COVID-19 patients is higher than in the above-mentioned contexts. Yet, it reports less of depression prevalence than the depression rates reported in the studies of Kisely et al. [49] and Tung et al. [61].

Depression is among the five most debilitating disorders, and it is predicted to be one of the key challenges in the developed nations by 2030 [36]. Depression is often conceptualized as a set of negative symptoms such as negative mood, negative cognition, and avoidance behaviors. Accordingly, effective psychological therapies for depression, such as cognitive–behavioral therapy and interpersonal therapies, have focused on reducing or improving these negative aspects by shifting dysfunctional beliefs, identifying avoidance behaviors, and resolving interpersonal problems [62, 63].

The results of this study show that the prevalence of depression, anxiety and stress in the Hospital staff caring for the COVID-19 patients is high. Our work has also discussed the importance of treating these disorders and their potential impact on all aspects of a medical worker’s life. Therefore, interventions are necessary to improve such workers’ lifestyles, through regular monitoring of potential depression, anxiety and stress disorders, and to reduce the associated side effects. In addition, since depression, anxiety and stress can be prevented in the first place, and can also be controlled and treated if they advance, it is necessary to offer full training to Hospital staff on depression, anxiety and stress, and how they could be prevented. Moreover, it is essential to control and treat these disorders as early as possible, and through timely diagnosis. Due to the high prevalence of stress, anxiety and depression within front-line healthcare workers caring for COVID-19 patients, it is recommended that physicians be more attentive to the symptoms of these disorders. Furthermore, media should provide related information with the aim of raising people's awareness to prevent delayed diagnosis.

## Limitations

One of the limitations of this research is the lack of uniform reporting in the selected studies, the non-uniformity of the methodologies. Moreover, due to the limitation in finding articles from different continents, and the lack of uniform distribution of articles in different geographical locations, subgroup analysis was not performed on different continents, or ethnic groups. Also, given that the COVID-19 pandemic started in China, most of the studies reviewed include articles from this country, and this situation affects the generalization of results worldwide.

## Conclusions

The results of this study clearly demonstrate that the prevalence of stress, anxiety and depression within front-line healthcare workers caring for COVID-19 patients are high. Therefore, the healthcare authorities, and decision-makers, nationally and internationally, should take measures to reduce these disorders in Hospital staff treating the COVID-19 patients. This increases the productivity of the Hospital staff, speeds up the measures to control the pandemic, and provides more effective treatment procedures for the COVID-19 patients.

## Data Availability

Datasets are available through the corresponding author upon reasonable request.

## Abbreviations

SARS: Severe Acute Respiratory Syndrome
MERS: Middle East Respiratory Syndrome
WHO: World Health Organization
PHEIC: Public Health Emergency of International Concern
ICTV: International Committee on Virus Classification
STROBE: Strengthening the Reporting of Observational studies in Epidemiology
PRISMA: Preferred Reporting Items for Systematic Reviews and Meta-Analysis
